# Clinical characteristics and prognosis of pediatric *Listeria monocytogenes* meningitis based on 10-year data from a large children’s hospital in China

**DOI:** 10.1128/spectrum.03244-23

**Published:** 2024-01-26

**Authors:** Xueqian Xia, Lingyu Zhang, Hao Zheng, Xiaoling Peng, Li Jiang, Yue Hu

**Affiliations:** 1 Department of Neurology, Children’s Hospital of Chongqing Medical University, Chongqing, China; 2 Department of Neonatology, Women’s Hospital, School of Medicine, Zhejiang University, Hangzhou, China; 3 Guangdong Provincial Key Laboratory of Interdisciplinary Research and Application for Data Science, BNU-HKBU United International College, Zhuha, China; 4 Ministry of Education Key Laboratory of Child Development and Disorders, Chongqing, China; 5 National Clinical Research Center for Child Health and Disorders, Chongqing, China; 6 China International Science and Technology Cooperation Base of Child Development and Critical Disorders, Chongqing, China; 7 Chongqing Key Laboratory of Pediatrics, Chongqing, China; London Health Sciences Centre, London, Canada

**Keywords:** *Listeria monocytogenes *meningitis, children, clinical characteristics, treatment

## Abstract

This study aims to summarize the clinical characteristics and prognosis of *Listeria monocytogenes* (LM) meningitis in children in Chongqing, China. A retrospective analysis of the clinical data and follow-up results of 20 LM meningitis patients admitted to the Children’s Hospital of Chongqing Medical University (CHCMU) from January 2012 to December 2022 was performed. The LM meningitis incidence rate was between 0 and 14.3 per 100,000 persons. The median age at onset was 8.98 months. There were five neonate cases, which all had perinatal abnormalities. Seven non-neonatal cases (7/15, 46.7%) had a documented history of contaminated food intake. One case had primary immunodeficiency. The most common symptoms were fever (20/20), altered consciousness (19/20), and vomiting (15/20). Seven cases had seizures, eight cases had cranial nerve involvement, eight cases had positive Babinski sign, and 10 cases had positive meningeal signs. The most common complications were hyponatremia (6/20), hypokalemia (6/20), respiratory failure (5/20), subdural effusion (3/20), and hydrocephalus (2/20). Treatment primarily involved monotherapy or combination therapy with meropenem (15/20) and ampicillin (10/20). Fifteen cases were treated with monotherapy or combination therapy using vancomycin. Twelve cases were successfully followed up from 10 months to 9 years and 6 months, and all had favorable long-term outcomes. LM meningitis incidence in children is low and with nonspecific clinical manifestations. Strengthening food hygiene and safety education, and avoiding infections during pregnancy are important to prevent LM infection in neonates and high-risk individuals. Meropenem and ampicillin are the preferred treatments. Early diagnosis and treatment can improve prognosis.

**IMPORTANCE:**

The incidence of LM meningitis is extremely low, and there is currently no standardized treatment. We conducted a retrospective analysis of ten years of data from CHCMU regarding diagnosed LM meningitis cases, aiming to provide clinical evidence for the diagnosis and treatment.

## INTRODUCTION

*Listeria monocytogenes* (LM) is an ubiquitous Gram-positive bacillus that can grow at low temperatures and withstand low pH and high salt concentrations, which is responsible for listeriosis, a foodborne disease transmitted through the consumption of contaminated food, especially ready-to-eat food products, such as fish and fishery products, meat products from bovines or pigs, fruits and vegetables, and cheeses from sheep milk (1). When LM-contaminated food has been consumed, the LM passes through the intestinal epithelium and enters the target organs via the lymphatic system and bloodstream. Furthermore, the bacterium can cross the blood–brain barrier and placental barrier (2). Newborns, elderly people, and immunocompromised people are particularly susceptible to LM infection. The clinical symptoms of listeriosis are fever, gastroenteritis, and invasive infections, including sepsis and meningitis (3–5).

Listeriosis is a globally recognized foodborne disease. Although the LM infection incidence rate is generally low, the mortality rate can be as high as 24%–62% without timely treatment (6, 7). The incidence of listeriosis was estimated to be 0.3 per 100,000 persons in 2021. Of the 148 infected patients, 140 (95%) required hospitalization, with a mortality rate of 25% (8). In the United Kingdom, New Zealand, and Israel, the perinatal listeriosis incidence rates were 3.4/100,000 (9), 12.3/100,000 (10), and 5–25/100,000 (11), respectively. In Southeast Asia, Singapore reported a perinatal listeriosis incidence rate of 4.8/100,000 (12). As a result, developed countries such as the United States and France, among others, have established surveillance systems for LM infection, officially designating listeriosis as a reportable disease (13). In China, LM infection incidence was low and only sporadic cases were reported. From 2008 to 2017, only 759 cases of LM infection were reported. The most common presentation was septicemia (49%) and central nervous system (CNS) infection (25%). The overall case fatality rate was 18%, with a higher rate among neonatal patients (73%) (14). The regional variations may be related to the serotypes of LM prevalent in the area and to local dietary habits. Whole-genome sequencing and metagenomics provide valuable insights for implementing scientific strategies in the prevention and control of foodborne illnesses (15).

Compared to other meningitis-causing bacterial pathogens, such as *Streptococcus pneumoniae*, *Neisseria meningitidis*, and *Haemophilus influenzae*, LM has a higher propensity to invade the central nervous system (16, 17). More than 90% of newborns with LM infection developed meningitis (4, 18). Non-neonatal children have lower LM meningitis incidence compared to neonates. In Danish children (aged 1 month to 17 years), the 2000–2017 incidence rate of LM meningitis was 0.024/100,000 children (19).

In the present study, we conducted a retrospective analysis of clinical data and the follow-up results of children diagnosed with LM meningitis at the Children’s Hospital of Chongqing Medical University (CHCMU), Chongqing, China, from January 2012 to December 2022, to provide clinical evidence for the diagnosis and treatment of LM meningitis.

## MATERIALS AND METHODS

### Inclusion criteria

The inclusion criteria were age <18 years; LM-positive blood and/or cerebrospinal fluid (CSF) culture, combined with meningeal irritation signs or CSF leukocytosis (>10 × 10^6^/L), decreased CSF glucose (<0.4 g/L), CSF glucose/blood glucose ratio <0.6, or elevated CSF protein (>0.45 g/L) (20).

Other intracranial infectious diseases were ruled out conclusively after admission to our hospital for examination and treatment. All patients were followed for ≥9 months from discharge and when the disease had entered the sequelae phase. Informed consent was obtained from the guardians of all participating patients.

### Exclusion criteria

Patients were excluded from the analysis if they had viral encephalitis, tuberculous meningitis, or cryptococcal meningitis, or other potential CNS diseases.

### Definitions

Early-onset neonatal LM meningitis was defined as age at onset within 7 days; whereas, late-onset LM meningitis referred to age at onset between 7 and 28 days (21). The LM meningitis hospitalization mortality rate was defined as the number of patients who died during hospitalization divided by the total number of patients. The annual LM meningitis incidence rate was defined as the number of diagnosed cases per 100,000 admissions per year from January 2012 to December 2022 (22). The effective antibiotic duration referred to the total duration of an antibiotics regimen that included any of the following antibiotics: amoxicillin, ampicillin, penicillin G, sulfamethoxazole, moxifloxacin, meropenem, linezolid, vancomycin, and gentamicin (23).

### Cultivation and identification of LM

The LM in this study were isolated and identified from blood cultures and CSF cultures. Venous blood was collected and injected into blood culture bottles and was cultured using the BacT/ALERT 3D fully automated blood culture system. CSF was directly inoculated onto blood agar plates, chocolate agar plates, and enrichment broth, and then placed in a CO_2_ incubator for cultivation. After incubation, the bacteria from blood and CSF were identified to the species level using VITEK Mass Spectrometry.

According to the M45 guidelines for infrequently isolated or fastidious bacteria (24), LM is a bacterium for which susceptibility testing was not typically performed. Susceptibility testing is only conducted in patients whose antibiotic treatment is ineffective. Therefore, in this study, susceptibility testing was not conducted. The minimum inhibitory concentration of meropenem is ≤0.25, and ampicillin or amoxicillin is ≤2 in M45 guideline. Meropenem is used empirically before the culture results are available (24). After the culture results are available, the antibiotic treatment regimen will not be adjusted when the clinical symptoms improve. For the patients with poor antibiotic effectiveness, the antibiotic will be adjusted to monotherapy or combination therapy with ampicillin, which is considered as the first-line antibiotic for LM (23, 25).

### Prognostic evaluation

The short- and long-term patient prognoses were evaluated with the Glasgow Outcome Score (GOS). The short-term evaluation was based on the GOS at discharge, and the long-term evaluation was based on the GOS after a follow-up period of >9 months. The GOS was graded as follows (26): 5: good recovery, might have slight physical or mental impairments; 4: moderate disability, unable to resume previous activities; 3: severe disability, conscious but unable to independently carry out daily activities; 2: vegetative state; 1: death. A GOS of 5 was defined as a good prognosis, while a GOS of 1–4 was defined as a poor prognosis. Patients with a GOS clinical outcome score of 5 were included in the favorable prognosis group, while those with scores of 1–4 were included in the unfavorable prognosis group.

### Statistical analysis

The data were processed and analyzed with SPSS Statistics 26.0. In the descriptive statistics, count data were described with frequencies and proportions, and measurement data were expressed using the mean and standard deviation or median (interquartile range [IQR]) depending on the normality of the measurement data, which was evaluated by the kurtosis coefficient and skewness coefficient. Count data were analyzed using the Mann-Whitney U test, while the measurement data were analyzed using Fisher’s exact test. Variables demonstrating statistical significance were included in multivariate logistic regression analyses (*P* < 0.05). The missing values were dropped from the analysis.

## RESULTS

### Clinical presentation and auxiliary examinations

This study included 20 patients diagnosed with LM meningitis at CHCMU from January 2012 to December 2022 (Table 1). The annual incidence rate of LM meningitis in children was between 0 and 14.3 per 100,000 from 2012 to 2022 (2012: 14.3, 2013: 0, 2014: 3.6, 2015: 1.7, 2016: 4.7, 2017: 1.5, 2018: 1.4, 2019: 1.3, 2020: 4.6, 2021: 6.2, and 2022: 1.3, respectively). The patients’ age at onset ranged from 4 hours to 7 years (median age: 8.98 months). Five neonatal LM meningitis cases had perinatal abnormalities, and four cases (4/5, 80%) had onset within 24 hours after birth and presented with meconium-stained amniotic fluid and respiratory distress. One neonatal case was associated with maternal antenatal fever and positive amniotic fluid culture for LM. Two neonatal cases were premature infants born at 32 + 4 and 32 + 6 weeks of gestation. Hearing screening for all five cases revealed no abnormalities.

**TABLE 1:** Analysis of risk factors associated with antibiotic duration in children with LM meningitis

Clinical manifestations and auxiliary examinations		Antibiotic duration ≥ 4 weeks (*n* = 11)	Antibiotic duration < 4 weeks (*n* = 9)	*P*-value
Sex				0.653
Male		4/20 (20%）	5/20 (25%）	
Female		7/20 (35%）	4/20 (20%）	
Age at onset (days)				1.0
≤6		3/20 (15%)	2/20 (10%)	
7–28		0	0	
＞28		8/20 (40%)	7/20 (35%)	
Season at onset				
Spring (March–May)		4/20 (20%)	2/20 (10%)	0.642
Summer (June–August)		3/20 (15%)	3/20 (15%)	1.0
Autumn (September–November)		2/20 (10%)	2/20 (10%)	1.0
Winter (December–February)		2/20 (10%)	2/20 (10%)	1.0
Gestational age^*a*^ (weeks)				1.0
<37		1/5 (20%)	1/5 (20%)	
≥37		2/5 (40%)	1/5 (20%)	
Birth weight^*a*^				1.0
<2.5 kg		1/4 (25%)	1/4 (25%)	
≥2.5 kg		2/4 (50%)	0	
Mode of delivery^*a*^				1.0
Vaginal birth		1/5 (20%)	0	
Cesarean section		2/5 (40%)	2/5 (40%)	
Perinatal abnormalities^*a*^		3/5 (60%）	2/5 (40%)	
History of contaminated food and drink		3/20 (15%)	4/20 (20%）	0.642
Duration of illness		53.1 ± 4.72	29.9 ± 4.34	
＜2 weeks		0	1/20 (5%）	
2–4 weeks		0	1/20 (5%）	
≥4 weeks		11/20(55%）	7/20 (35%）	
Average number of cumulative antibiotic use		5.64 ± 0.364	3.78 ± 0.364	0.002
Average duration of antibiotic use (days)		46.1 ± 4.17	21.3 ± 2.15	
Effective antibiotic use duration (days)		40.5 ± 4.15	19.8 ± 1.88	
Effective antibiotic use duration/total antibiotic use duration (%)		92.5 (22.06）	100 (11.11）	0.112
Clinical manifestations and physical examination	Fever	11/20 (55%)	9/20 (45%)	
	Fever peak (°C)	39.4 ± 0.27	39.3 ± 0.27	0.656
	Fever duration (days)	19 (22）	9 (12）	0.175
	Vomiting	8/20 (40%)	7/20 (35%)	1.0
	Diarrhea	5/20 (25%)	1/20 (5%)	0.157
	Altered state of consciousness	10/20 (50%)	8/20 (40%）	1.0
	Hypothermia	0	1/9 (11.1%）	0.45
	Convulsive seizures	4/20 (20%）	3/20 (15%）	1.0
	Increased fontanelle tone	7/20 (35%）	1/20 (5%）	0.028
	Abnormal muscle tone	4/20 (20%）	2/20 (10%）	0.0642
	Yellowish skin	2/20 (10%)	1/20 (5%）	1.0
	Stiff neck (with suspicion)	5/20 (25%)	5/20 (25%）	1.0
	Positive Babinski sign (with suspicion)	3/20 (15%)	5/20 (25%）	0.362
	Abnormal pupillary light reflex	4/20 (20%）	4/20 (20%）	1.0
	Respiratory failure/respiratory distress	2/20 (10%)	3/20 (15%）	0.617
Ancillary examination				
Initial blood leukocyte count (10^9^/L)		19.9 ± 3.94	18.6 ± 2.78	0.766
	＜4.3	3/20 (15%)	0	0.218
	4.3~14.2	1/20 (5%）	4/20 (20%）	0.127
	＞14.2	7/20 (35%）	5/20 (35%）	1.0
Initial neutrophil percentage (%)		60.9 ± 7.63	79.6 ± 3.90	0.046
Initial hemoglobin (g/L)		96 (29）	119 (34）	0.025
＜90		1/20 (5%）	0	1.0
Initial platelet count (10^9^/L)		404 (689）	234 (216.5）	0.152
＞450		5/20 (25%）	1/20 (5%）	0.157
＜100		1/20 (5%）	1/20 (5%）	0.197
Albumin (g/L)	＜35	5/20 (25%）	6/20 (30%）	0.406
ALT or AST (U/L)	＞50	5/20 (25%）	2/20 (10%）	0.374
Potassium ion (mmol/L)	＜3.5	2/20 (10%）	4/20 (20%）	0.336
	＞5.5	4/20 (20%）	0	0.094
Sodium ion (mmol/L)	＜132	2/20 (10%）	4/20 (20%）	0.336
	＞149	0	2/20 (10%）	0.189
Disease-onset C-reactive protein (mg/L)	<8	3/20 (15%)	1/20 (5%）	0.591
	8–30	5/20 (25%)	4/20 (20%）	1.0
	>30	3/20 (15%)	4/20 (20%）	0.642
Disease-onset procalcitonin (ng/dL)	<1.1	8/20 (40%)	4/20 (20%）	0.362
	1.1–10	2/20 (10%)	3/20 (15%）	0.617
	>10	1/20 (5%）	2/20 (10%）	0.566
Disease-onset CSF white blood cell count (10^6^/L)		230 (496）	568 (2275）	0.503
	＜100	2/20 (10%)	3/20 (15%）	0.617
	100–500	5/20 (25%)	1/20 (5%）	0.157
	500–1,000	1/20 (5%）	2/20 (10%）	0.566
	＞1,000	3/20 (15%)	3/20 (15%）	1.0
Disease-onset CSF glucose concentration (mmol/L)	＜1.11	0	3/19 (15.8%）	0.058
	1.11–2	6/19 (31.6%）	5/19 (26.3%）	1.0
	＞2	5/19 (26.3%)	0	0.045
Disease-onset CSF protein concentration (g/L)	＜1	5/20 (25%)	2/20 (10%）	0.374
	1–2	4/20 (20%）	3/20 (15%）	1.0
	＞2	2/20 (10%)	4/20 (20%）	0.366
Disease-onset CSF synchronized glucose ratio	≤0.4	1/2 (50%)	4/6 (66.7%)	1.0
Last CSF white blood cell count before discharge (10^6^/L)		43 (175)	52 (72)	0.740
	＜15	7/17 (%）	3/17 (%）	0.35
	15–20	1/17 (%）	0	1.0
	≥20	2/17 (%)	4/17 (%）	0.362
Pathology	Positive blood culture only	4/20 (20%）	5/20 (25%）	0.653
	Positive CSF culture only	5/20 (25%)	4/20 (20%）	1.0
	Positive blood and CSF cultures	2/20 (10%)	0	0.479
Imaging abnormalities		4/20 (20%）	2/20 (10%）	0.642
	Subdural effusion	2/20 (10%)	1/20 (5%）	1.0
	Hydrocephalus	1/20 (5%）	1/20 (5%）	1.0
	Intracranial/subarachnoid hemorrhage	1/20 (5%）	0	1.0
	Softening of the brain	1/20 (5%）	0	1.0
	Brain atrophy	2/20 (10%)	1/20 (5%）	1.0
Electroencephalogram abnormalities				0.559
	Background slowing	1/14 (7.1%）	6/14 (42.9%）	0.029
	Atypical discharge	3/14 (21.4%）	0	0.192
Neurologic complications		4/20 (20%）	4/20 (20%）	1.0
	Subdural effusion	2/20 (10%)	1/20 (5%）	1.0
	Intracranial/subarachnoid hemorrhage	1/20 (5%）	0	1.0
	Brain infarction/softening/atrophy	1/20 (5%）	1/20 (5%）	1.0
	Brain hernia	0	1/20 (5%）	0.45
	Hydrocephalus	1/20 (5%)	1/20 (5%）	1.0
Adjuvant therapy				
	Subdural puncture	2/20 (10%）	1/20 (5%）	1.0
	Subdural incision and drainage	1/20 (5%）	1/20 (5%）	1.0
	V-P shunt	1/20 (5%）	0	1.0
	Ommaya reservoir placement	1/20 (5%）	0	1.0
	Ventilator-assisted	3/20 (15%）	2/20 (10%）	1.0
	Component blood transfusion	2/20 (10%）	2/20 (10%）	0.319
	Gammaglobulin	7/20 (35%）	4/20 (20%）	0.653
	Glucocorticoids	3/20 (15%)	2/20 (10%)	1.0

^*a*^ Gestational age and birth weight were only counted for neonatal patients in both groups. AST, aspartate aminotransferase; ALT, alanine aminotransferase.

Among the 15 non-neonatal patients, eight cases (8/15, 53.3%) were aged >1 year, and one had primary immunodeficiency. The onset season of six non-neonatal patients (6/15, 40%) was summer (spring: 4, summer: 6, autumn: 2, winter: 3, respectively). Seven of the 15 non-neonatal patients (46.7%) had a history of consuming contaminated food (3: meat, 4:cold delicacies).

### Treatment

**Fig 1:**
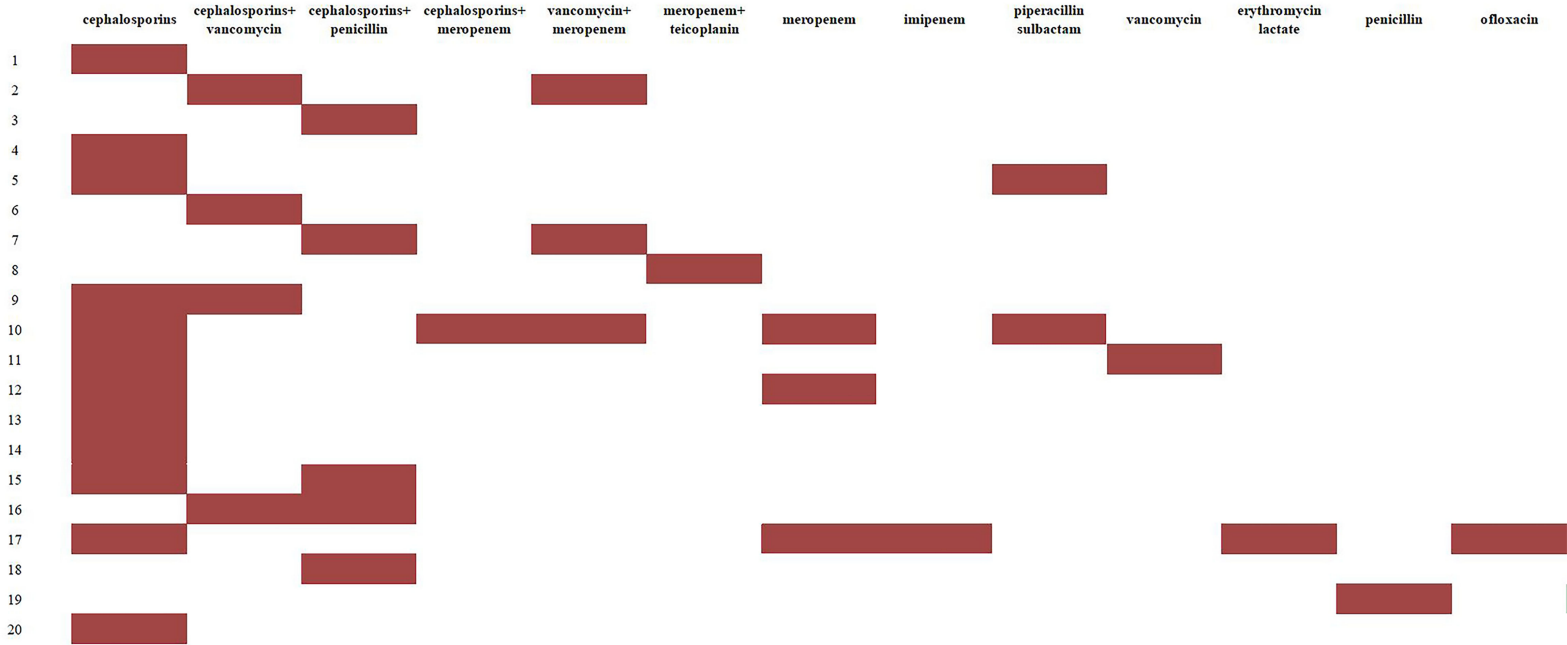
The antibiotic treatment regimens before positive cultures. *x* axis, antibiotic regimens; *y* axis, patient number.

**Fig 2:**
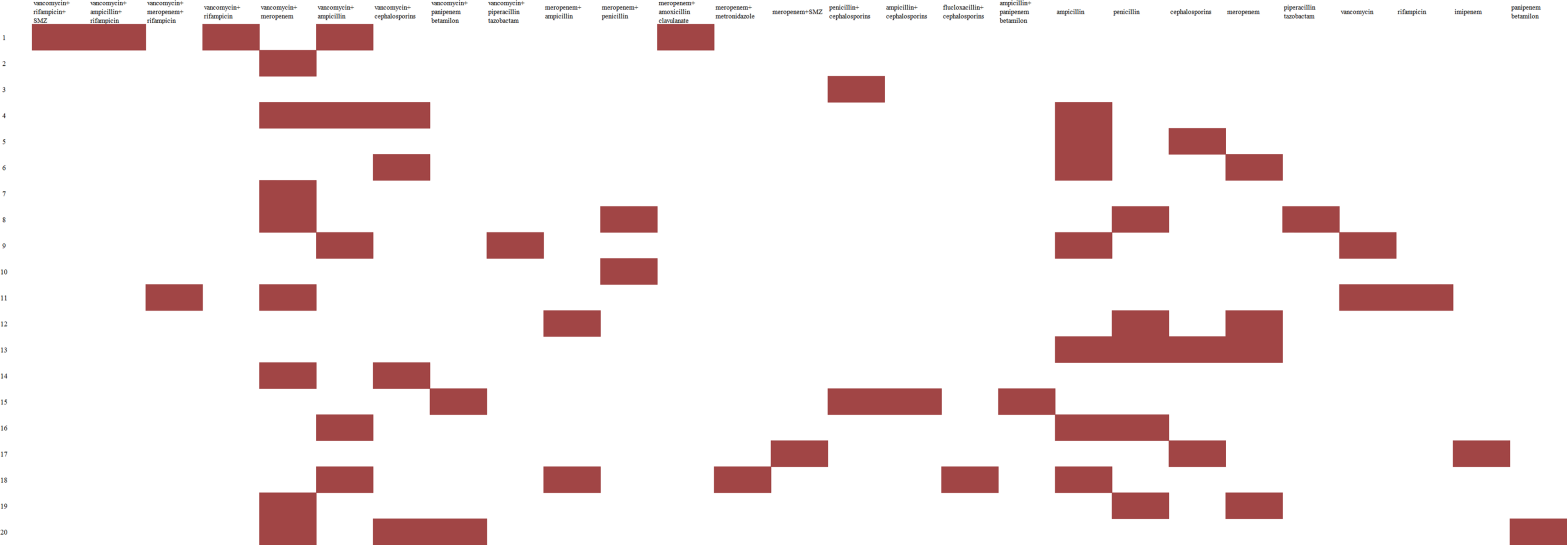
The antibiotic treatment regimens after positive cultures. *x* axis, antibiotic regimens; *y* axis, patient number; SMZ, compound sulfamethoxazole.

Antibiotic susceptibility testing and LM serotyping were not performed for any patient, and empirical antibiotic therapy was administered (Fig. 1). The cephalosporins were the most commonly used empirical antibiotics before culture results were available (60%, 12/20). Once the culture results were obtained, the most common empirical antibiotic regimen was vancomycin combined with meropenem (40%, 8/20) and monotherapy with ampicillin (35%, 7/20) (Fig. 2). Six cases empirically received meropenem before culture results were available, with clinical improvement observed in five cases. Therefore, these patients did not immediately switch antibiotics upon a positive culture result. For 10 cases with subsequent poor antibiotic response, antibiotic therapy was switched to ampicillin as monotherapy or in combination, based on a patient’s condition and guidelines. Considering the effectiveness of the previous antibiotics or infection severity, 15 patients (15/20, 75%) received monotherapy or combination therapy with vancomycin.

Univariate analysis suggested that the number of cumulative antibiotic types, increased bregma tension, peripheral blood neutrophil percentage at onset, peripheral blood hemoglobin count at onset, CSF glucose level >2 mmol/L at onset, and electroencephalogram background slowing were related with antibiotic treatment >4 weeks (*P* < 0.05). However, the multivariate regression analysis identified no statistically significant variables.

### Prognosis

Two patients (2/20, 10%) died during hospitalization, who were both non-neonatal patients. Six patients (6/20, 30%) were lost to follow-up in the long term. Among the 12 patients successfully followed up, the follow-up duration ranged from 10 months to 9 years and 6 months, and all had a favorable long-term prognosis.

## DISCUSSION

Under normal conditions, LM is cleared by the immune response upon invasion. However, in people with low or defective immunity, LM multiplies in the body and causes central nervous system LM infection through the gastrointestinal tract (27, 28): (i) LM enters the CNS through the choroid plexus epithelial cells and causes meningitis; (ii) LM reaches the brain parenchyma through cerebral capillary endothelial cells and causes brain parenchymal lesions; (iii) LM-infected macrophages cross the blood–brain barrier through the cerebral arteries and result in encephalitis or brain abscesses; and (iv) LM invades the CNS through the peripheral nerves.

LM infection causes about 4% of community-acquired bacterial meningitis in patients over 16 years and about 1.5% of bacterial meningitis in newborns (29, 30). In the present study, the annual incidence rate of LM meningitis in children was between 0 and 14.3 per 100,000. The median age at onset was 8.98 months, and there were five neonatal cases. Over 90% of human infections with *Listeria monocytogenes* are foodborne, resulting from the consumption of raw or inadequately cooked food contaminated with *Listeria*, or through cross-contamination (31). The consumption of contaminated food was deemed the main cause of LM infection. The prevalence of LM in food in Chongqing was 0.5% (32), and the consumption of Chinese cold dishes increased the infection risk (33). In the present study, 7 of the 15 non-neonatal patients (46.7%) had a history of consuming contaminated food, and 1 patient had primary immunodeficiency. Among the 20 patients, 12 (60%) had an onset in the spring or summer. LM infections in the gastrointestinal tract were more common in the summer, indicating seasonal variation (34), which differed from the study in the Netherlands (35). In pregnant people, the LM infection risk when consuming contaminated food was 16–18 times higher compared to the general population, as maternal immunity is reduced during pregnancy (36). It is recommended to implement effective control and monitoring measures in the final stages of processing for ready-to-eat foods, with a priority on conducting quantitative risk assessment studies for LM in cooked meat products and Chinese-style cold dishes to reduce potential hazards.

Furthermore, pregnant people infected with LM transmit the bacteria to the fetus and newborn through the placenta and birth canal, respectively. The transmission routes include ascending placental–fetal infection, ascending amniotic fluid infection, and hematogenous placental–fetal infection. In the present study, all five cases of neonatal LM meningitis had perinatal abnormalities. Four neonatal cases were classified as early-onset LM infection.

LM infections in the CNS have diverse and nonspecific clinical manifestations. The most common symptoms include fever, headache, neck stiffness, and altered state of consciousness. Other possible manifestations include seizures, cranial nerve palsies, impaired consciousness, and brainstem, cerebellum, and extrapyramidal system involvement. LM infections can affect the meninges and brain parenchyma, resulting in meningitis, meningoencephalitis, and in a minority of cases, rhombencephalitis or brain abscesses (3, 37–39). The main clinical features observed in the present study were fever (20/20, 100%), vomiting (15/20, 75%), and altered state of consciousness (19/20, 95%). The main complications were hyponatremia (6/20, 30%), hypokalemia (6/20, 30%), respiratory failure (5/20, 25%), etc.

Most LM infections feature elevated peripheral blood leukocyte count, C-reactive protein, and procalcitonin levels, and increased erythrocyte sedimentation rate. Some patients might also develop bacteremia and hyponatremia (40, 41). In the present study, the peripheral blood inflammatory indicators demonstrated different degrees of change, which were consistent with previous research findings (40, 41). The changes indicated the importance and feasibility of blood routine dynamic follow-up during clinical treatment to assess disease severity in bacterial meningitis. The laboratory parameters of CSF in LM meningitis (cell count, protein level, glucose level) were similar to that of other bacterial meningitis. However, increased intracranial pressure (8/20, 40%) was more prominent in LM meningitis. As it is difficult to distinguish LM meningitis from tuberculous meningitis, cryptococcal meningitis, and other CNS bacterial infections based on clinical manifestations and routine CSF examination, the diagnosis standard relies on CSF and blood culture. In the present study, the early CSF culture positivity rate for LM was 55% (11/20 cases), and 11 cases (11/20, 55%) also had LM bacteremia. Therefore, early completion of blood culture before antibiotic use can increase the pathogen detection rate. Given its non-invasiveness and visual representation, the results of neuroimaging (computed tomography, magnetic resonance imaging) are also important indicators for assessing disease severity. In the present study, six patients (6/20, 30%) had abnormal neuroimaging findings.

The LM meningitis incidence rate is extremely low (9–12, 14, 19), and there is currently no standardized diagnostic and treatment protocol. Due to the drug resistance of LM to first-generation quinolones, fosfomycin, and third-generation cephalosporins, the European Society of Clinical Microbiology and Infectious Diseases (ESCMID) guidelines recommend amoxicillin, ampicillin, or penicillin G as the first-line treatment for LM meningitis (23). Additionally, trimethoprim/sulfamethoxazole, quinolones, and rifampicin can also be used to treat LM infection, but vancomycin is not recommended as empirical therapy for LM infection (5). Our institution did not routinely conduct antimicrobial susceptibility testing for LM (24). In the present study, the main treatment regimens were monotherapy or combination therapy involving meropenem (15/20, 75%) and ampicillin (10/20, 50%).

With the exception of the two deaths, the follow-up outcomes demonstrated that infection was effectively controlled in the remaining 18 patients, and the 12 patients successfully followed up had a good long-term prognosis. LM was mainly type 1/2a in China, and the mortality rate of type 1/2a was lower than type 4b (42–44). This may explain why the mortality rate in our study was only 10%. The antibiotic treatment duration was 2–4 weeks, or at least 1 week after fever subsidence. In immunocompromised patients, the treatment duration can be extended to 6 weeks (45). In the present study, 11 cases (11/20, 55%) received antibiotic treatment for >4 weeks. The univariate analysis suggested six factors might be associated with antibiotic treatment for >4 weeks (*P* < 0.05). However, the multivariate regression analysis revealed no statistically significant variables. Due to the limited sample size and potential bias, further studies are needed. The use of steroids as part of the empirical treatment for bacterial meningitis, especially in patients with suppurative meningitis and brain parenchymal involvement, is controversial (3, 46, 47). The ESCMID guidelines recommend discontinuing steroids in patients with LM infection (23). In the present study, five patients received steroids as adjunctive treatment, and although one patient was lost to follow-up, the remaining patients had good short- and long-term prognoses.

In summary, LM meningitis has an extremely low incidence rate, and there is currently no standardized treatment protocol. Strengthening food hygiene and safety education, and avoiding infections during pregnancy are important measures to prevent LM infection in newborns and high-risk individuals. In clinical practice, vigilance against possible LM infection is necessary for patients with suppurative meningitis, especially those with brain parenchymal involvement. Early completion of CSF and blood microbiological examinations and prioritizing neuroimaging evaluations are crucial. Disease control should also be prioritized, followed by antibiotic regimen adjustment based on pathogen identification and susceptibility testing. Empirical treatment with meropenem and ampicillin is recommended. Early diagnosis and treatment can lead to improved prognosis.

## Data Availability

The study does not involve the genome sequences. The authors confirm that the data supporting the findings of this study are available within the article. The original clinical data involving the patients' privacy will not be disclosed publicly. Any additional data are available on request from the corresponding author.
